# The London Hospital

**Published:** 1902-03-15

**Authors:** 


### THE LONDON HOSPITAL.

### FIGURES FOR THE YEAR 1901.

The Hon. Sydney Holland, who has again been elected chairman of the house committee, presided at the Quarterly Court of Governors of the London Hospital on the 5th inst., at which was presented a report dealing with the completed figures for the past year, as well as with the work of the three months just ended.

I do not think it is generally realised, he said, in moving the adoption of the report, that at this hospital we treat twice as many cases as any of the other hospitals except one, and very nearly twice as many as even that one. If properly appreciated our figures would, I am sure, have an astonishing effect on those to whose minds they were brought home. Our total of in-patients treated in 1901 was 13,364, a slight increase as compared with the 13,234 in the previous year. The largest number of patients in the wards in one day during the whole year was 760, and, since the demand for beds is very great, only the most urgent cases can be admitted. It can scarcely be realised by anyone not actually in the hospital what it means to have 760 really bad cases in one day. The actual average number daily in the wards during the year was 678—one of the highest averages ever reached. . As showing what that means, I might mention that at a great institution like St. Thomas's the average is 300. The mean residence of each patient was rather less than 20 days—19·45 to be precise. Few people know that the London is one of the largest, if not the largest, children's hospital in England. Last year we treated 3,547 children under 12 years of age. How heavy the work has been may be readily understood from the fact that the number of major operations in the theatres and operating rooms was no less than 2,469, or 8 a day; while the total number of anæsthetics administered in the hospital was 8,591, or 24 a day. The applications in the receiving room for first aid were heavy, numbering 166,379; and 2,641 maternity cases were visited outside by the maternity assistants under the supervision of the obstetrical registrar or the resident accoucheur. The total number of patients treated in the out-patient department, practically all of them being sent from the receiving room was:—accident cases, 18,833—that is, over 50 accidents a day; general medical and surgical cases, 38,625; and in the special departments, such as the skin, aural, dental, and ophthalmic, 17,097 were treated. In the aural department in particular there were 2,895 cases, and the committee have now elected Mr. Hunter Tod as an additional assistant aural surgeon.

### The Finsen Cure for Lupus.

Our new department for the treatment of lupus by the light cure was also kept busy during the year, the total number of attendances being 18,649. We have a waiting list of 260 patients who will be treated in their turn. The pressure, however, on this hospital is not so heavy as it was, as other hospitals both in London and in the provinces have found it necessary to instal this light, and they give people the opportunity of being treated without coming to this hospital. We shall try to bring this before Her Majesty the Queen that she may know how great a benefit has followed on her introduction of Finsen's light cure into this country. Those complete the figures for the year, and they constitute a record which we may all be proud of having helped to carry out. I might mention here that we have very reluctantly been obliged, in consequence of the prevalence of small-pox, to stop all visitors to patients. It is a painful necessity, and we shall not maintain it any longer than we can help.

### Progress of the Alterations.

The governors will have noticed on entering the hospital that considerable progress has been made in the building operations during the quarter. The main corridor from the centre lobby to the west wing is now complete, and the committee trust that it meets with the full approval of the governors. The main staircase at the west end is not yet quite complete, but the corridors on the first and second floors are now open for traffic. The residents' rooms on the second floor are ready for occupation, and we hope that within a month the theatres will also be ready for use. We shall then have a range of five of the finest theatres in England, fitted up simply and inexpensively but most effectively, and the work of the hospital will be both facilitated and improved in dealing with the enormous number of surgical cases we have. And here I might say that I think it the greatest pity attacks, such as I saw published the other day, should be made on the Jews. The Jews have always been good friends to this hospital, and these five theatres were given by a Jew, anonymously, and the only condition made was that they should be open to Jews and Christians alike. Another attack I deprecate, this time on ourselves, is that made last week at the Stepney Borough Council, because we had not proper room for witnesses in the Coroner's Court. We know quite well it wants improvement, and we hope as soon as we begin to get the benefit of the new buildings, to make satisfactory arrangements there as elsewhere. But we are trying to rebuild the hospital without closing a single ward, and necessarily that means inconvenience more or less to everybody. We are quite sure had the gentleman known the facts he would not have spoken as he did.

### Other Improvements in Progress.

The electric light is being gradually installed, and in all the newer parts of the building the light is already on and the wiring in the old parts of the building is being proceeded with. The east wing improvements are making good progress. The whole of the ground floor wards "Gloucester" and "Cambridge" have been reorganised, and the walls are now being covered with opalite. The progress will be made upwards with this wing, and when the third floor is being done the whole of the old "Cotton" wards will be rebuilt, and above them again will be constructed first-rate modern kitchens. We shall be able to open the out-patients' department in the first week in August. As soon as this is done progress can be made at once with the rearrangement of the west wing, a most important branch of the work, as we shall then be able to get on with the new Jewish wards, the present Jewish wards being rearranged as maternity wards. The plans and machinery have been finally approved by the committee for the new laundry which will be built on the site of the old Brewers' Almshouses. On January 25th the Lord Mayor (who is a vice-president, and was for many years on the House Committee) and the Sheriffs visited the hospital in state, and expressed the greatest satisfaction at the progress being made; one of the Sheriffs afterwards sent a donation of 60 guineas for himself and wife. Dr. Salaman, an old student of the hospital, has been appointed assistant to Dr. Bulloch in the pathological department.

### Deaths on the Nursing Staff.

It is with the deepest regret that we have to inform the governors of the fact that we have suffered the loss through death of two of the most valuable members of the hospital nursing staff. Each of these nurses died as the result of a very serious operation. Nurse Loweth, who was well known to many of the governors as Nurse "Harrison," had been on the staff and had been highly valued as an able nurse for

16 years. Latterly she occupied the important position of nurse in charge of the Nurses' Home sick room. The other nurse whose death we have to deplore is Miss Annie Brewster, better known as Nurse "Ophthalmic," who has been here for no less than 20 years. These deaths have cast a gloom over this last quarter. The hospital can ill afford to lose two such devoted women from its staff. Miss Wamsley, who was many years Sister "Cotton," has been appointed matron of the Herman de Stern Convalescent Home at Felixstowe. This building is now completed, and will be occupied early in the spring. A sub-committee who visited this home recently and went over it spoke in enthusiastic terms of the building as "a splendid building erected on an ideal site." It will be remembered that Baroness de Stern gave £60,000, of which £10,000 was to build the home and £50,000 to endow it.

### Donations and Legacies.

Amongst the donations which have been received during the past quarter, attention must be called, in the first instance, to the King Edward's Fund contribution of £5,000. It will be remembered that this sum is promised annually, on the condition that £100,000 is spent on improvements. Then, too, the Hospital Saturday Fund has given us £1,045, the largest sum ever received from them. We have also received £1,000 from Lady Tate, two anonymous donations of £1,000, and a further donation of £600. £300 from the Baroness Burdett-Coutts, £250 from the Duchess of Bedford, £100 from Wm. Cadbury, Esq., £100 from Hugh Martineau, and a sum of £100 collected especially for the light department by Miss Millington. Mr. Hora, a vice-president, has already helped the Samaritan Society very largely, and it will be remembered that it is now called the Marie Celeste Samaritan Society. This year, in addition to the £6,000 before given, he has added another £1,000 to the endowment of the Marie Celeste ward, so-called in memory of his wife. The new maternity ward will be called the Marie Celeste ward, but till it is built one of the wards, in what will be the isolation block, has been so christened. In legacies, we have received £500 from Mrs. Peppin, £350 on account of the late Mrs. Edgell Hunt, and £200 from Richard Lewis. It may be mentioned that the total amount received from legacies during the year was £11,442 15s. 6d., a sum which is rather below our average, and we would point out to the governors that we have to depend very much on these legacies, and we hope that all friends of the hospital will do their best to secure as full benefit as possible on every occasion in this direction.

Mr. Shepherd said he had been connected with the hospital for 30 years, and had always felt a pride in it; but he had never had cause to be so proud of it as now. Another Governor thought the committee were deserving of the greatest credit.

The report was adopted, and the proceedings were terminated by an invitation from the Chairman to those who cared to inspect the progress of the alterations, of which a good many availed themselves, and were greatly impressed with the magnitude and excellence of the work.

